# Cumulative Pesticides Exposure of Children and Their Parents Living near Vineyards by Hair Analysis

**DOI:** 10.3390/ijerph18073723

**Published:** 2021-04-02

**Authors:** Elisa Polledri, Rosa Mercadante, Dario Consonni, Silvia Fustinoni

**Affiliations:** 1 Department of Clinical Sciences and Community Health, Università degli Studi di Milano, Via S. Barnaba, 8, 20122 Milan, Italy; elisa.polledri@unimi.it (E.P.); rosa.mercadante@unimi.it (R.M.); 2 Fondazione IRCCS Ca’ Granda Ospedale Maggiore Policlinico, Via S. Barnaba, 8, 20122 Milan, Italy; dario.consonni@policlinico.mi.it

**Keywords:** pesticides, hair, biomonitoring, vineyards, child

## Abstract

The aim of the present work was the application of hair biomonitoring to investigate exposure to pesticides in children and their parents residing in a vineyard area. Thirty-three children and 16 parents were involved in the study. Hair samples were self-collected before and after the application season (PRE- and POST-EXP samples). Information on study subjects and the use of pesticides in the area were obtained. Thirty-nine pesticides were analyzed by liquid chromatography tandem mass spectrometry, and thirty-one pesticides were quantifiable in at least one hair sample. Most frequently detected pesticides were chlorpyrifos, cycloxidim, dimethomorph, metalaxyl, spiroxamine, and tetraconazole. From PRE-EXP to POST-EXP the percentage of quantification and/or the concentration of pesticides increased; the concentration was typically in the low pg/mg hair range with comparable levels in children and parents. An inverse correlation was found between the total exposure to pesticides in POST-EXP hair samples and the distance between home and the treated fields (Spearman ρ = −0.380, *p* = 0.01). The results of this study show that the majority of the study pesticides were measured in the hair of subjects living in the close proximity of treated vineyards, supporting the determination of pesticides in hair for the purpose of biomonitoring cumulative exposure in the general population.

## 1. Introduction

A pesticide consists of several different components that prevents, destroys, or controls a harmful organism (“pest”) or disease, or protects plants or plant products during production, storage, and transport [1]. Before a pesticide can be commercialized on the European market, its active substance needs to be approved according with the Regulation No 1107/2009 on Plant Protection Products, the Regulation No 396/2005 on maximum residue levels in food (MRL), and the Directive 2009/128/EC on sustainable use of pesticides [1,2,3]. The whole process is set to ensure safe use of pesticides in the EU regarding human health and environmental sustainability.

Nevertheless, the large amount of these chemicals voluntarily spread in the environment, the past use of unsafe and/or persistent pesticides, and the recent controversies between public bodies has cast doubt on the integrity of the authorization process in public opinion. Recently, European citizens have asked for the process of authorization to be rethought to focus on public health, the environment, and sustainable agriculture, and to ensure that decision-makers make data available to the civil society and the scientific community [4,5]. This concern is particularly pronounced in citizens residing in rural areas where the use of pesticides takes place, especially regarding the risk for health of the more vulnerable ones, such as children. This fragile category may be exposed through inhalation of pesticide drift and/or volatilization from applications made in nearby crop fields, by parental take-home exposures and by residential use [6,7], by diet—particularly fruits and vegetables [8,9,10]—and by house dust [11,12,13].

Vineyards are a cultivar in which the use of pesticides is particularly intense; annually in Northern Italy about 25 kg/h of active substances are applied in treated vineyards, while between 1 and 10 kg/h are used in the other crops [14]. Pesticides can be used in the different stages of vine growth: firstly herbicides are applied to keep the ground clean; during spring (in which rains can be abundant) fungicides are applied to fight molds that can infest leaves and/or inflorescences; finally, in the last part of the growing period, in which grapes are ripening, insecticides are used against insects.

Evaluation of exposure is a crucial step for risk assessment; among the different techniques, biological monitoring plays a relevant role, as it is able to take into account all the possible routes of exposure, all sources, and the individual behavior [15,16]. However, biomonitoring may provide unrealistic data when based on pesticide metabolites in spot urine samples, which account only for short-term exposure, disregarding cumulative and aggregate exposures.

Recently the use of hair as a matrix for biomonitoring of the exposure to pesticides has been investigated [17,18]. The main features of hair biomonitoring are the large data acquisition window (from weeks to months depending on the length of the sample), the non-invasiveness, easy collection and transport, and the lack of a requirement for special conditions for conservation of the sample. Moreover, the determination of pesticides in hair greatly hastens the process of biomarkers discovery as it focuses on the determination of several unmetabolized pesticides, easily measurable with available multi-residues analytical assays, without the need for investigating human metabolisms.

Until now, the research on biomonitoring of exposure to pesticides in the general population using hair focused mostly on persistent pesticides, such as DDT and its metabolites [11,19,20,21,22,23]; conversely, few studies investigated currently used pesticides in the hair of the general population [24,25], including children [17], and only some recent studies applied a multi-residual method to assess the exposure to mixtures of pesticides [22,24,25]. Recently, we developed and validated an analytical assay based on liquid chromatography tandem mass spectrometry to assess the presence of several currently used pesticides in hair, characterized by a short persistence in the environment [26].

The aim of the present work was the application of hair biomonitoring to investigate aggregate and cumulative exposure to 39 currently used pesticides in children and their parents residing in a rural area located in Northern Italy, extensively cultivated with wine-grape.

## 2. Materials and Methods

### 2.1. Study Population

The study was conducted in 2018 in a vineyard area of the province of Treviso, Veneto, Italy. The study was co-organized with the citizen association ColtiviAMOfuturo, area Grappa, Asolo, Montello and Piave. Among the association members, there are several families living in villages surrounded by an intensely cultivated wine area and worried about exposure to pesticides for their children and themselves. The association itself recruited the study subjects among its members, which participated on a voluntary base. All subjects were informed about the aim of the study; parents signed a written informed consent for themselves and for their children.

A sample collection kit with instruction for hair sampling and a questionnaire was given to each participant. Parents self-collected hair samples from their children and themselves after having carefully washed hands. A lock of hair was cut, as close as possible to the root, in the occipital region of the head, using fine scissors; the lock had a diameter of approximatively 5–8 mm and a variable length. The lock of hair was attached with paper masking tape on a sampling sheet that indicated the direction of the hair (root-tip) and was stored at room temperature in the dark in a paper envelope. Subjects were instructed to keep a lock of occipital hair in case of haircut between PRE- and POST-EXP sampling. The treatment of vineyards with herbicides, fungicides and insecticides was performed from May to September. Hair samples were collected in May, before the application season (PRE-EXP) and again in August, at the end of the application season (POST-EXP).

A self-administered questionnaire was used to gain personal information (i.e., gender, age, weight, hair color, and smoking habit) and some possible determinants of exposure (i.e., the last haircut, the hair washing frequency, hair treatments, the distance between home and the nearest vineyard, and the consumption of vegetables and fruits grown in the area).

Samples and questionnaires were collected and delivered to the laboratory in September. Once in the laboratory, samples were kept in the dark at room temperature until analysis.

### 2.2. Pesticides Selection

The pesticides to be tested were chosen with the following criteria:

- Certainly Used in the study Area (CUA) = the citizen association, with the consultancy of an agronomist, expert on the cultivars of the area, provided an initial list of 9 pesticides certainly used in the study area (chlorpyrifos, cycloxidim, dimethomorph, mandipropamid, meptylninocap, metalaxyl, pyraclostrobin, spiroxamine, and tetraconazole).
- Probably Used in the study Area (PUA) = Another 12 pesticides that were probably used in the study area, as approved in the protocol of the consortium of the vineyard farmers [27], were added to this list (azoxystobin, boscalid, cyprodinil, fenamidone, fludioxonil, indoxacarb, iprovalicarb, metrafenone, penconazole, pyrimethanil, quinoxyfen, and zoxamide).
- Probably Used in the Surroundings (PUS) = Another 9 pesticides probably used in the surroundings, that were not authorized by the farmers’ consortium, but widely used in other vineyards, were also added (bupirimate, chlortoluron, cyproconazole, diuron, etofenprox, imidacloprid, metobromuron, terbuthylazine, and tebuconazole).
- Persistent Pesticides Not Authorized (PPNA) = Finally, 9 persistent pesticides not authorized by the European Commission [28], but widely used in the past and with a high persistence in the environment, were included in the list (atrazine, bitertanol, carbendazim, linuron, methabenzthiazuron, metoxuron, monolinuron, sebuthylazine, and simazine).

The complete list of 39 measured pesticides, together with their CAS number, their agrochemical category, their approval status according with the EU regulation and/or the farmers consortium protocol, are reported in Table 1.

All the chemicals used in this study are reported in a previously published method [26].

### 2.3. Hair Preparation and Extraction

Sample preparation was performed as previously described [26]. Briefly, a segment of hair of 3 cm length, measured starting from the root, and with an approximate weight of 50–100 mg, was added to 2 mL of ultra-pure water and vortexed at room temperature to remove contaminants on the hair surface. The length of the hair segment was chosen to cover approximatively the last three months of exposure to pesticides. In the case of the POST-EXP sample, this was the period in which the pesticides were spread in the vineyards. The aqueous solution was then completely removed and analyzed to control the presence of the analytes; no presence of pesticides was detected. Rinsed hair was dried at 60 °C for one hour, cut, and introduced into a 2 mL cryogenic tube (Eppendorf, Safe-Lock tube, Milan, Italy). The tube was placed in a grinding jar and was cooled down in a bath with liquid nitrogen for about 10 min; then the sample was milled (MM400, Retsch Italy, Torre Boldone, Italy). A known amount of about 50 mg of hair powder was transferred into a glass vial. Hair powder was added with 2 mL CH_3_CN and IS solution; the vial was sealed and the sample was extracted at 45 °C for 3 h with a horizontal shaker with a rotatory vibration. An aliquot of the extract was completely dried. 50 µL of CH_3_CN were used to reconstitute and 5 µL of this sample were analyzed by liquid chromatography tandem mass spectrometry (LC-MS/MS). The precision of the overall process was <4% for all the analyzed pesticides, while the extraction efficiency could only be evaluated as a relative extraction, comparing different extraction media and conditions in hair samples of exposed donors, for which quantifiable levels of almost all analyzed pesticides could be detected, and taking the most effective condition as the reference (100%) [26].

### 2.4. LC-MS/MS Analysis of Pesticides

The analysis were performed with a high performance liquid chromatography (LC, Agilent Technologies, Cernusco Sul Naviglio, Italy) equipped with an Acquity UPLC HSS T3 column (100 mm length, 2.1 mm internal diameter, 1.8 µm particle size, Waters, Sesto San Giovanni, Italy). The column was kept at 40 °C with a flow rate of 200 µL/min, using a linear gradient with two mobile phases: the A phase was composed by 5 mM ammonium formate in water with 0.1% of formic acid, while the B phase was composed by 5 mM ammonium formate in methanol with 0.1% of formic acid. The LC system was interfaced with a hybrid triple quadrupole/linear ion trap mass spectrometer (QTRAP 5500; Sciex, Monza, Italy) equipped with an electrospray ionization source (ESI), operated in scheduled selected reaction monitoring (sSRM) mode. The two most intense sSRM transitions were recorded for each native analyte; the most intense transition was used for quantitation, and the other one was used for qualification [26]. For each isotopically labelled standard, the most intense ion transition was recorded. The Analist^®^ software (version 1.6.3; Sciex, Monza, Italy) was used for setting up the method and the batches for analysis, while MultiQuant™ software (version 3.0.8664.0; Sciex, Monza, Italy) was used for quantification.

Together with the hair extracts, calibration solutions (0, 1, 2, 5, 10, 50, 100, 500, 1000, 5000, and 10,000 ng/L), and quality control solution (QC, 5 and 500 ng/L, low- and high-QC, respectively) were analyzed, with a precision, estimated as RSD%, <10% and an accuracy between 93 to 109% of the spiked concentrations. Linear regression curves were used to quantify pesticides concentrations in the extract (ng/L); these were than converted into concentrations in the hair samples (pg/mg hair) taking into account the weight of the hair sample and the extraction volume [26]. The two isomers of dimethomorph and cyproconazole were summed and considered together. Limit of quantification (LOQ) of the investigated pesticides was in the range of 0.04–0.20 pg/mg, as reported in Table 1.

### 2.5. Statistical Analysis

The concentration of pesticides in hair was classified as quantifiable or not quantifiable, based on comparison with the LOQ.

For quantifiable pesticides, data were analyzed either as dichotomous (i.e., values below or above the LOQ) or quantitative variables (samples with pesticide concentration ≥LOQ). The total exposure to pesticides (Σfmol_pest_/mg hair) was obtained by the sum of all measured pesticides, previously transformed in fmol.

We made two types of comparisons, across groups (unpaired data) and before–after (paired data). For unpaired data we calculated the Fisher exact test (dichotomous variables) and the Wilcoxon (also known as Mann–Whitney) rank-sum test (quantitative variables). For paired dichotomous data we calculated (a) the McNemar exact test; and (b) the differences of quantitation (POST-EXP minus PRE-EXP) with their 90% confidence intervals (CI) [29,30]. For paired quantitative data, we calculated (a) the Wilcoxon signed-ranks test; and (b) the geometric mean ratios (GMR, POST-EXP/PRE-EXP) and their 90% CI, because distributions were log-normal, as usual.

Correlations between the exposure to pesticides, both considering single molecules and the total exposure, and possible determinants of exposure were investigated with Spearman’s rank correlation coefficient rho (ρ).

Statistical analysis was performed using the SPSS 25.0 package for Windows (SPSS Inc., Chicago, IL, USA) and Stata 16 (StataCorp. 2019, College Station, TX, USA). Forest plots for before–after comparisons (frequency differences and GMRs) were produced with the Stata “metan” command. A *p* ≤ 0.05 was considered statistically significant.

## 3. Results

### 3.1. Study Population and Hair Samples

In Table 2 selected characteristics of study subjects and hair samples are summarized. Forty-nine subjects were initially recruited in the study, of which 33 were children and 16 were parents. Most of the participating parents were females (75%), and 70% of the involved children were male. For 3 children insufficient hair amount was obtained both in PRE- and in POST-EXP samples, so they were not further considered. A total of 46 subjects, collecting 42 PRE-EXP samples, 7 samples during the application season, and 45 POST-EXP samples, was the final study group. Pair samples, i.e., samples obtained from the same individuals in PRE-EXP and POST-EXP sampling, were 27 for children and 14 for parents. The majority of study subjects consumed vegetables and fruits grown in the area of residence, and they all lived very close to the vineyards (maximum distance 400 m).

### 3.2. Pesticides in Hair

Out of 39 measured pesticides, 8 were never detected in any hair sample and were not further included in the analysis. These pesticides were fenamidone (belonging to the PUA group), bupirimate, chlortoluron, etofenprox (PUS group), linuron, monolinuron, sebuthylazine, and simazine (PPNA group). Results of the 31 pesticides detected in hair of study subjects are reported in Table 3, grouped by sampling time and by study group (children and parents). Data are given as number and percentage of samples above LOQ, and as median, minimum and maximum of concentrations (pg/mg hair). Seven subjects had haircuts during the application season and collected an intermediate sample, in addition to the PRE- and POST-EXP samples. For them, the POST-EXP results are given as the sum of pesticides in POST-EXP sample and in the intermediate sample.

Considering PRE-EXP samples, in children, 26 out of 39 pesticides were quantifiable at least in one subject; similarly, in parents 24 out of 39 pesticides were quantifiable at least in one subject. Considering both children and parents, the median levels of pesticides in hair ranged from 0.05 to 3.29 pg/mg hair (for zoxamide and chlorpyrifos, respectively). Total exposure to pesticides (Σfmol_pest_/mg hair) in PRE-EXP samples ranged from 12.5 to 16.8 fmol/mg hair, for children and parents, respectively.

Considering POST-EXP samples, in both children and parents, 31 pesticides were detectable at least in one sample, with the only exception of atrazine that was below LOQ in children. In positive samples, the median levels of pesticides were in the range of 0.06 to 5.28 pg/mg hair (for indoxacarb and chlorpyrifos, respectively). Altogether, total exposure to pesticides in POST-EXP samples was 68.7 and 82.6 fmol/mg hair, for children and parent, respectively.

In both children and parents, pesticides with the highest concentrations in hair were chlorpyrifos, cycloxidim, dimethomorph, and spiroxamina, all belonging to the group of CUA.

**Figure 1:**
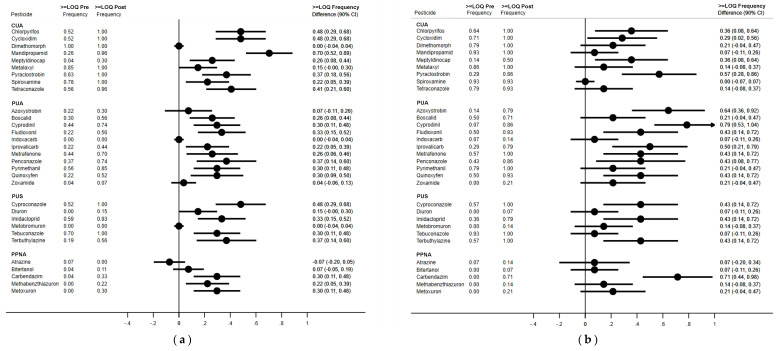
(**a**) Children: frequency of quantitation of pesticides in paired PRE-EXP and POST-EXP hair samples (N = 27) and difference (90% CI) between the frequencies of quantitation. CUA = Pesticides Certainly Used in the Area; PAU = Pesticides Probably Used in the Area; PUS = Pesticides Possibly Used in the Surroundings; PPNA = Persistent Pesticides Not Authorized. (**b**) Parents: frequency of quantitation of pesticides in paired PRE-EXP and POST-EXP hair samples (N = 14) and difference (90% CI) between the frequencies of quantitation. CUA = Pesticides Certainly Used in the Area; PAU = Pesticides Probably Used in the Area; PUS = Pesticides Possibly Used in the Surroundings; PPNA = Persistent Pesticides Not Authorized.

The comparison among frequency of quantitation showed that, for several pesticides, the percentage of quantifiable samples increased from PRE- to POST-EXP. In particular, the frequency of quantitation increased for 22 pesticides in children, and 15 pesticides in parents (see Table 3 and Figure 1a,b).

**Figure 2:**
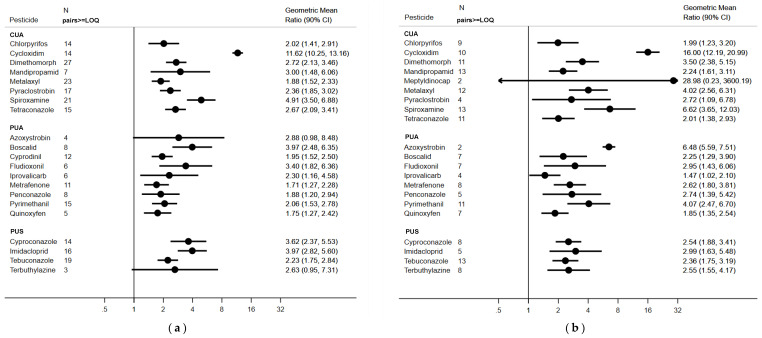
(**a**) Children: geometric mean of the difference (90% CI) between the concentration of pesticides in hair in POST-EXP and PRE-EXP samples. N pairs ≥ LOQ indicates the number of paired samples with a detectable concentration of pesticides available for the comparison. CUA = Pesticides Certainly Used in the Area; PAU = Pesticides Probably Used in the Area; PUS = Pesticides Possibly Used in the Surroundings. (**b**) Parents: geometric mean of the difference (90% CI) between the concentration of pesticides in hair in POST-EXP and PRE-EXP samples. N pairs ≥ LOQ indicates the number of paired samples with a detectable concentration of pesticides available for the comparison. CUA = Pesticides Certainly Used in the Area; PAU = Pesticides Probably Used in the Area; PUS = Pesticides Possibly Used in the Surroundings; PPNA = Persistent Pesticides Not Authorized.

In paired samples, i.e., pesticides ≥LOQ in both PRE- and POST-EXP (number of pairs ranged from 2 to 27), we found that for almost all pesticides the concentrations at the end of the application season increased. In particular, the concentrations were significantly higher for 15 pesticides for children, and 16 pesticides for parents out of 20 considered pesticides (see Figure 2a,b).

**Figure 3:**
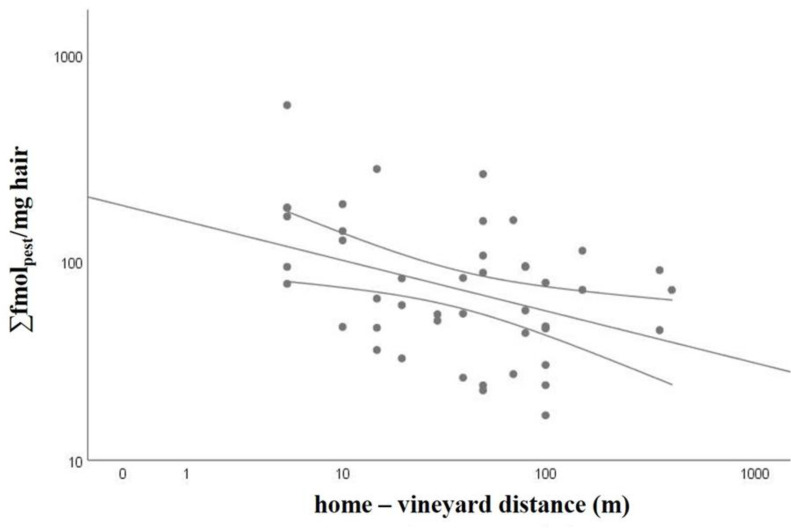
Scatter plot and linear regression line between total exposure to pesticides (∑fmol_pest_/mg hair) in POST-EXP samples of all study subjects and the distance between home and cultivated vineyard (m).

Considering the correlations between exposure to pesticides and the possible determinants of exposure, only the distance between the residence and the treated vineyards was correlated with the total exposure to pesticides (Σfmol_pest_/mg hair) in POST-EXP samples. The scatter plot in Figure 3 shows the negative correlation, with Spearman ρ = −0.380 and *p* = 0.010. The correlation was significant also separately considering pesticides in CUA and PUA (Spearman ρ = −0.364 and −0.368, *p* = 0.013 and 0.014, respectively), but not considering those in the PUS and PPNA groups. No correlation was found for other possible determinants of exposure, such as hair color, the last haircut, the hair washing frequency, hair treatments, and diet based on fruit and vegetables locally grown.

## 4. Discussion

In the present study, hair was used to assess the exposure to 39 pesticides in children and their parents living close to vineyards. The majority of pesticides were incorporated into hair and increased during the application season.

In this work, the selected pesticides were chosen based on the probability of use, with nine pesticides certainly applied (CUA) and another 12 pesticides approved by the farmers’ consortium protocol for the use in the vineyards (PUA) (Table 1). Unsurprisingly, these pesticides were the most frequently found and those with the highest concentrations (Table 3). In particular, for the CUA group, chlorpyrifos was always quantified in POST-EXP hair samples and was the pesticide with the highest concentration (median up to 5.28 and maximum level up to 33.8 pg/mg hair); this is explained considering that chlorpyrifos is the only insecticide certainly applied in the vineyards. Among the herbicides, both cycloxidim and mandipropamid were from the CUA group: they were consistently found in the large majority of samples, but the percentage of quantification and the concentration were higher for cycloxidim than for mandipropamid, with medians up to 3.12 vs. 0.39 pg/mg hair, respectively. This suggests a larger use for the first than for the second herbicide, even if we cannot exclude that other determinants, such as a different absorption, metabolism, and storage rates can also explain this result. Among the seven fungicides belonging to the CUA group, we found the highest concentrations for spiroxamine and dimethomorph, with median and maximum levels up to 3.83 and 37.7 pg/mg hair and 1.27 and 18.8 pg/mg hair, respectively. These results suggest a higher use of these fungicides in comparison with the others.

For the PUA group, the highest level of pesticide in hair was detected for the fungicide pyrimethanil, with a median and maximum concentrations of 0.42 and 48.6 pg/mg hair; this substance was approved in the protocol of farmers’ consortium, although it was not in the list of pesticides known to be applied. Considering the PUS group, the insecticide imidacloprid, not approved by the consortium, but approved by the EU regulation and widely used for other types of grapes, was found in 90% of children’s POST-EXP hair, with median and maximum concentrations of 0.20 and 32.7 pg/mg hair. Moreover, exposure to imidacloprid could be associated with the use of new generation bait to fight beetles in domestic/indoor environments. Finally, it is relevant to note that pesticides in the PPNA group were not found (linuron, monolinuron, sebuthylazine, and simazine) or were found in very small percentage/concentration (atrazine, bitertanol, methabenzthiazuron, metoxuron). For the latter pesticides, both their frequency of quantitation and their concentration did not increase during the application season. These evidences support their ubiquitous presence in the environment due to a past use and their long persistence. The only exception was for carbendazim, a fungicide not approved by the EU [28], that was found in 33% of children’s and 73% of parent’s POST-EXP hair samples. Rather than an illegal use of this product, the result may be explained by the use of thiophanate-methyl, an EU approved fungicide which breaks down to carbendazim, both in the human body and in the environment [28]. Overall, our findings indicate a legal use of pesticides by vineyard farmers, in accomplishment of the consortium internal protocol and the EU regulations, and an environmental diffusion with a consequent exposure of the rural residents.

The design of the study, including the collection of hair samples before and after the application season, was meant to evaluate the capability of hair to reflect the cumulative exposure during the growing season. The same design was previously applied to investigate exposure to organophosphates, terbuthylazine, penconazole and tebuconazole, and a mixture of 27 pesticides in agricultural workers [22,31,32,33]. In the present study, similarly to the previous ones, an accumulation of pesticides during the growing season was found, but major novelties were introduced as the enlargement of the investigated pesticides and the application of the protocol to the rural general population, including children.

An increase in the frequency of quantification was found for 22 pesticides in children (Figure 1a) and for 15 pesticides in parents (Figure 1b). For dimethomorph, a CUA group pesticide, no increase in the detection frequency was observed, given the fact that it was quantified in almost all samples in both PRE- and POST-EXP samples (Table 3, Figure 1a,b).

Figure 2 reports the comparison between the concentration of pesticides in hair in POST- and PRE-exposure samples. The number of pairs samples varies from 2 to 27, because only pairs samples with quantifiable concentrations of pesticides could be included. Nevertheless, the analysis showed a general increase in POST-EXP samples for almost all the included pesticides, with a mean increase of 3-fold in children (ranging from 1.71 for metrafenone to 11.65 for cycloxidim), and a mean increase of 4-fold in parents (ranging from 1.47 for iprovalicarb to 16.0 for cycloxidim). The increase, as expected, follows this trend: CUA > PUA > PUS.

Pesticide levels were comparable in children and their parents (Table 3). Only for cycloxidim and mandipropamid parents showed significantly higher levels in POST-EXP samples (*p* = 0.01 and *p* = 0.03, respectively). This suggests that childhood behaviors, such as playing in the garden and/or ingesting ground dust, were not a significant source of additional exposure.

Negative correlations between the exposure to pesticides, either expressed as total pesticides or as CUA or PUA, in POST-EXP hair and the home-to-treated fields distance were found (Figure 3). This supports the use of hair for quantitative biomonitoring of cumulative pesticide exposure in people living near the treated areas and confirms our previous results, showing a relationship between the number of treatments/quantity of applied pesticides during the season and the concentration in hair for occupationally exposed subjects [32,33].

Among studies investigating pesticides in the hair of the general population, it is worth mentioning a recent work measuring 140 pesticides and metabolites in the hair of 311 French pregnant women [24]. Comparing this study with the present work, we note that common pesticides, such as carbendazim, tebuconazole, azoxystrobin, diuron, boscalid, and imidacloprid, were similar for both quantitation frequency and concentration. Differently, a previous study of our group [33], investigating pesticides in agricultural workers and in a few agricultural relatives, reported higher concentrations of pesticides in the hair of agricultural relatives in comparison with rural residents of the present study. Those may be explained with a higher exposure of agricultural relatives, due to take-home exposure from occupational settings.

Specific limitations of the present study are the small number of investigated subjects and the fact that they were citizens worried for their exposure to pesticides, so our results cannot be regarded as representative of the exposure to pesticides of the Italian general population living near vineyards. Moreover, we assumed that pesticide applications in the vineyards during the growing season are responsible for the difference in the exposure levels detected in POST- vs. PRE-EXP hair samples. However, other mechanisms such as the difference in food habits in summer and the higher temperatures that increase the volatility of pesticides might also have influenced the level of exposure over the study period. In addition, the stability over time of the pesticides in the hair matrix was only assumed, not proved; since the POST-EXP samples are more recent, degradation over time might also explain part of our results.

Several other general limitations of hair biomonitoring were listed in the Summary report “Hair analysis panel discussion: exploring the state of the science”, promoted by the U.S. Agency for Toxic Substances and Disease Registry [34]. Among others, there are the difficulty distinguishing between external contamination and real internal dose, the absence of data for predicting adverse effects in health through hair measurements, and the lack of interpretation criteria, such as reference values. In fact, the development of biomonitoring exposure to organic chemicals in hair analysis is promising, but is still at its initial stage and therefore it must be limited to research purposes. In spite of the limitations associated with the hair biomonitoring, we believe that the potential advantages of using hair as a matrix capable of integrating mixture chemical exposures over months are relevant and worth further investigation. Moreover, it can be foreseen that the use of new technologies, such as mass spectrometry, and further studies on the biology and toxicokinetic of hair will be beneficial to collect additional data for building a frame for the future interpretation of hair biomonitoring.

Among the strengths of this study there are the double hair sampling that allowed to evaluate the difference between pre- and post-application and to speculate about the cumulative exposure along the growing season, and the application of a validated multi-residue analytical assay with a high sensitivity, which allowed us to measure very tiny concentration of pesticides in non-professionally exposed subjects.

## 5. Conclusions

Our results showed that the majority of investigated pesticides was measured into the hair of rural children and their parents residing near a large area with vine cultivar. The increased quantitation frequency and concentration at the end of the season, and the inverse relationship between the total level of pesticides in hair and the home-to-crop distance, add new elements that support the use of hair biomonitoring for assessing aggregate and cumulative exposure to pesticides in the general population, including vulnerable categories, such as children.

## Figures and Tables

**Table 1:** Categories and list of measured pesticides, CAS number, agrochemical category, EU approval status, approval status according with the farmers’ consortium rules, and the analytical assay limit of quantification (LOQ).

Pesticides Group	Pesticides	CAS	Agrochemical Category	EU Status	Approved by the Farmers’ Consortium	LOQ (pg/mg Hair)
Pesticides Certainly Used in the study Area (CUA)	Chlorpyrifos	2921-88-2	Insecticide	Approved	Approved	0.08
	Cycloxidim	101205-02-1	Herbicide	Approved	Approved	0.08
	Dimethomorph	110488-70-5	Fungicide	Approved	Approved	0.04
	Mandipropamid	374726-62-2	Fungicide, Herbicide	Approved	Approved	0.04
	Meptyldinocap	131-72-6	Fungicide	Approved	Approved	0.04
	Metalaxyl	57837-19-1	Fungicide	Approved	Approved	0.04
	Pyraclostrobin	175013-18-0	Fungicide	Approved	Approved	0.04
	Spiroxamine	118134-30-8	Fungicide	Approved	Approved	0.08
	Tetraconazole	107534-96-3	Fungicide	Approved	Approved	0.04
Pesticides Probably Used in the study Area (PUA)	Azoxystrobin	131860-33-8	Fungicide	Approved	Approved	0.04
	Boscalid	188425-85-6	Fungicide	Approved	Approved	0.04
	Cyprodinil	121552-61-2	Fungicide	Approved	Approved	0.04
	Fenamidone	161326-34-7	Fungicide	Not Approved Max period of grace: 14/11/2019	Approved	0.04
	Fludioxonil	131341-86-1	Fungicide	Not Approved Max. period of grace: 31/10/2018	Approved	0.08
	Indoxacarb	173584-44-6	Insecticide	Not Approved Max. period of grace: 31/10/2018	Approved	0.04
	Iprovalicarb	140923-17-7	Fungicide	Approved	Approved	0.04
	Metrafenone	220899-03-6	Fungicide	Approved	Approved	0.04
	Penconazole	66246-88-6	Fungicide	Approved	Approved	0.04
	Pyrimethanil	53112-28-0	Fungicide	Approved	Approved	0.04
	Quinoxyfen	124495-18-7	Fungicide	Approved	Approved	0.04
	Zoxamide	156052-68-5	Fungicide	Approved	Approved	0.04
Pesticide Probably Used in the Surroundings (PUS)	Bupirimate	41483-43-6	Fungicide	Approved	Not approved	0.04
	Chlortoluron	15545-48-9	Herbicide	Not Approved Max. period of grace: 31/10/2018	Not approved	0.08
	Cyproconazole	94361-06-5	Fungicide	Approved	Not approved	0.04
	Diuron	330-54-1	Herbicide	Not Approved Max period of grace: 30/09/2018	Not approved	0.20
	Etofenprox	80844-07-1	Insecticide	Approved	Not approved	0.20
	Imidacloprid	138261-41-3	Insecticide	Approved	Not approved	0.04
	Metobromuron	3060-89-7	Herbicide	Approved	Not approved	0.20
	Terbuthylazine	5915-41-3	Herbicide	Approved	Not approved	0.04
	Tebuconazole	107534-96-3	Fungicide	Approved	Not approved	0.04
Persistent Pesticides Not Authorized (PPNA)	Atrazine	1912-24-9	Herbicide	Not Approved	Not approved	0.04
	Bitertanol	55179-31-2	Insecticide	Not Approved	Not approved	0.08
	Carbendazim *	10605-21-7	Fungicide	Not Approved	Not approved	0.08
	Linuron	330-55-2	Herbicide	Not Approved	Not approved	0.20
	Methabenzthiazuron	18691-97-9	Herbicide	Not Approved	Not approved	0.04
	Metoxuron	19937-59-8	Herbicide	Not Approved	Not approved	0.08
	Monolinuron	1746-81-2	Herbicide	Not Approved	Not approved	0.20
	Sebuthylazine	7286-69-3	Herbicide	Not Approved	Not approved	0.20
	Simazine	122-34-9	Herbicide	Not Approved	Not approved	0.20

* Carbendazim can also be found following the application of thiophanate-methyl, an EU approved pesticide that can breaks down to carbendazim both in the environment and in human body. CAS = Chemical Abstracts Service number.

**Table 2:** Personal characteristics of study subjects and number of hair samples.

			Children	Parents	Total
Subjects initially recruited, n			33	16	49
Subjects without valid hair samples, n			3	0	3
Subjects entering the study, n			30	16	46
PRE-EXP hair samples only, n			0	1	1
POST-EXP hair samples only, n			3	1	4
PRE-EXP + POST-EXP paired hair samples, n			27	14	41
Gender	n male (%)		21 (70%)	4 (25%)	25 (54%)
	n female (%)		9 (30%)	12 (75%)	21 (46%)
Mean age (minimum-maximum)			6 (1.5–16)	42 (35–51)	
Home-to-vineyards distance (m) Mean (minimum-maximum)			73 (5–400)	63 (5–400)	67 (5–400)
Consumption of vegetables grown in the study area, n (%)		Never	6 (20%)	3 (19%)	9 (20%)
		Rarely	4 (13%)	2 (12%)	6 (13%)
		Often	16 (54%)	4 (25%)	20 (43%)
		Usually	4 (13%)	7 (44%)	11 (24%)
Consumption of fruits grown in the study area, n (%)		Never	3 (10%)	3 (19%)	6 (13%)
		Rarely	8 (27%)	4 (25%)	12 (26%)
		Often	15 (50%)	4 (25%)	19 (41%)
		Usually	4 (13%)	5 (31%)	9 (20%)

**Table 3:** Summary of statistics of pesticides in hair in samples collected before (PRE-EXP) and after (POST-EXP) the application season. Results of 31 pesticides with at least one quantifiable sample are reported in pg/mg hair.

Pesticides Group	Pesticide		Children			Parents			Children vs. Parents	
			PRE-EXP	POST-EXP	*p* Value PRE- vs. POST-EXP ^a,b^	PRE-EXP	POST-EXP	*p* Value PRE- vs. POST-EXP ^a,b^	*p* Value PRE-EXP ^c,d^	*p* Value POST-EXP ^c,d^
CUA	Chlorpyrifos	N ≥ LOQ (%)	14 (52)	30 (100)	<0.001	9 (60)	15 (100)	0.06	0.75	na
		Median (min–max)	3.29 (2.07–7.41)	3.83 (0.79–21.9)	0.14	2.94 (2.41–28.2)	5.28 (1.41–33.8)	0.18	0.64	0.16
	Cycloxidim	N ≥ LOQ (%)	14 (52)	30 (100)	<0.001	11 (73)	15 (100)	0.12	0.21	na
		Median (min–max)	0.16 (0.10–0.32)	1.86 (0.58–4.37)	<0.001	0.18 (0.12–0.40)	3.12 (0.68–4.46)	<0.001	0.24	0.01
	Dimethomorph	N ≥ LOQ (%)	27 (100)	30 (100)	1.00	12 (80)	15 (100)	0.25	0.04	na
		Median (min–max)	0.30 (0.05–4.97)	1.06 (0.22–12.9)	0.001	0.39 (0.07–10.4)	1.27 (0.30–18.8)	0.04	0.66	0.62
	Mandipropamid	N ≥ LOQ (%)	7 (26)	28 (93)	<0.001	14 (93)	15 (100)	1.00	<0.001	0.55
		Median (min–max)	0.11 (0.06–0.27)	0.19 (0.06–3.36)	0.04	0.24 (0.06–0.78)	0.39 (0.09–1.76)	0.01	0.04	0.03
	Meptyldinocap	N ≥ LOQ (%)	1 (4)	10 (33)	0.01	3 (20)	8 (53)	0.06	0.12	0.22
		Median (min–max)	0.38	0.31 (0.08–10.8)	0.75	0.07 (0.05–0.14)	1.15 (0.14–7.10)	0.02	0.18	0.29
	Metalaxyl	N ≥ LOQ (%)	23 (85)	30 (100)	0.12	13 (87)	15 (100)	0.50	1.00	na
		Median (min–max)	0.24 (0.05–1.30)	0.42 (0.08–5.20)	0.02	0.18 (0.08–1.84)	0.51 (0.13–7.04)	0.01	0.75	0.26
	Pyraclostrobin	N ≥ LOQ (%)	17 (63)	30 (100)	0.002	4 (27)	13 (87)	0.008	0.05	0.11
		Median (min–max)	0.16 (0.06–0.66)	0.33 (0.09–1.37)	0.01	0.30 (0.13–0.45)	0.47 (0.07–1.58)	0.14	0.28	0.15
	Spiroxamine	N ≥ LOQ (%)	21 (78)	30 (100)	0.03	14 (93)	14 (93)	1.00	0.39	0.33
		Median (min–max)	0.92 (0.37–3.13)	3.83 (0.42–37.7)	<0.001	0.74 (0.13–1.73)	2.61 (0.63–30.1)	0.001	0.14	0.40
	Tetraconazole	N ≥ LOQ (%)	15 (56)	28 (93)	0.001	12 (80)	14 (93)	0.50	0.18	1.00
		Median (min–max)	0.14 (0.06–0.57)	0.30 (0.05–1.53)	0.001	0.20 (0.11–0.88)	0.42 (0.18–1.01)	0.02	0.06	0.34
PUA	Azoxystrobin	N ≥ LOQ (%)	6 (22)	11 (37)	0.69	2 (13)	11 (73)	0.004	0.69	0.03
		Median (min–max)	0.07 (0.06–0.19)	0.29 (0.05–0.77)	0.04	0.08 (0.06–0.11)	0.21 (0.05–0.73)	0.08	0.86	0.97
	Boscalid	N ≥ LOQ (%)	8 (30)	18 (60)	0.01	8 (53)	11 (73)	0.25	0.19	0.51
		Median (min–max)	0.08 (0.05–0.15)	0.20 (0.06–2.55)	0.004	0.14 (0.05–0.35)	0.30 (0.05–3.85)	0.13	0.24	0.43
	Cyprodinil	N ≥ LOQ (%)	12 (44)	22 (73)	0.008	2 (13)	13 (87)	0.001	0.05	0.46
		Median (min–max)	0.09 (0.04–0.21)	0.11 (0.05–0.71)	0.10	0.15 (0.06–0.24)	0.18 (0.05–0.57)	0.67	0.52	0.37
	Fludioxonil	N ≥ LOQ (%)	6 (22)	16 (53)	0.004	7 (47)	14 (93)	0.03	0.16	0.01
		Median (min–max)	0.21 (0.10–0.46)	0.29 (0.09–3.19)	0.29	0.24 (0.09–1.45)	0.25 (0.09–7.48)	0.91	0.47	0.76
	Indoxacarb	N ≥ LOQ (%)	0	1 (3)	1.00	1 (7)	2 (13)	1.00	0.36	0.25
		Median (min–max)		0.06	na	0.08	0.16 (0.12–0.21)	0.22	na	0.22
	Iprovalicarb	N ≥ LOQ (%)	6 (22)	14 (47)	0.03	5 (33)	11 (73)	0.01	0.48	0.12
		Median (min–max)	0.07 (0.06–0.41)	0.11 (0.05–0.70)	0.11	0.20 (0.06–0.34)	0.12 (0.06–0.77)	0.87	0.71	0.60
	Metrafenone	N ≥ LOQ (%)	12 (44)	21 (70)	0.04	8 (53)	15 (100)	0.03	0.75	0.02
		Median (min–max)	0.11 (0.05–0.33)	0.18 (0.05–1.20)	0.02	0.21 (0.05–0.83)	0.22 (0.10–3.43)	061	0.03	0.17
	Penconazole	N ≥ LOQ (%)	10 (37)	23 (77)	0.01	6 (40)	13 (87)	0.07	1.00	0.70
		Median (min–max)	0.07 (0.04–0.18)	0.11 (0.04–0.60)	0.02	0.06 (0.05–0.22)	0.14 (0.04–0.59)	0.11	0.87	0.83
	Pyrimethanil	N ≥ LOQ (%)	15 (56)	26 (87)	0.008	12 (80)	15 (100)	0.25	0.18	0.29
		Median (min–max)	0.16 (0.07–2.11)	0.23 (0.06–11.1)	0.36	0.15 (0.07–8.42)	0.42 (0.09–48.6)	0.03	0.64	0.07
	Quinoxyfen	N ≥ LOQ (%)	6 (22)	16 (53)	0.02	8 (53)	14 (93)	0.03	0.09	0.01
		Median (min–max)	0.08 (0.05–0.17)	0.10 (0.04–0.55)	0.48	0.06 (0.04–0.21)	0.15 (0.05–0.48)	0.04	0.56	0.20
	Zoxamide	N ≥ LOQ (%)	1 (4)	2 (7)	1.00	0	3 (20)	0.25	1.00	0.32
		Median (min–max)	0.05	0.16 (0.05–0.27)	0.48		0.12 (0.11–0.85)	na	na	0.56
PUS	Cyproconazole	N ≥ LOD (%)	14 (52)	30 (100)	<0.001	8 (53)	15 (100)	0.03	1.00	na
		Median (min–max)	0.07 (0.05–0.29)	0.26 (0.12–2.17)	<0.001	0.11 (0.06–0.23)	0.23 (0.19–0.68)	0.001	0.13	0.87
	Diuron	N ≥ LOQ (%)	0	5 (17)	0.12	0	2 (13)	1.00	na	1.00
		Median (min–max)		0.29 (0.23–4.47)	na		0.49 (0.22–0.75)	na	na	0.70
	Imidacloprid	N ≥ LOQ (%)	16 (59)	27 (90)	0.004	5 (33)	11 (73)	0.03	0.20	0.20
		Median (min–max)	0.09 (0.04–12.4)	0.20 (0.04–32.7)	0.08	0.31 (0.14–1.60)	0.37 (0.04–8.19)	0.78	0.14	0.69
	Metobromuron	N ≥ LOQ (%)	0	1 (3)	1.00	0	2 (13)	0.50	na	0.25
		Median (min–max)		0.21	na		0.25 (0.20–0.31)	na	na	1.00
	Terbuthylazine	N ≥ LOQ (%)	5 (19)	16 (53)	0.01	9 (60)	14 (93)	0.03	0.02	0.01
		Median (min–max)	0.06 (0.05–0.10)	0.12 (0.05–0.50)	0.02	0.08 (0.04–0.11)	0.17 (0.04–0.73)	0.02	0.25	0.43
	Tebuconazole	N ≥ LOQ (%)	19 (70)	30 (100)	0.008	14 (93)	15 (100)	1.00	0.12	na
		Median (min–max)	0.13 (0.04–0.91)	0.29 (0.04–1.63)	0.02	0.14 (0.05–0.68)	0.46 (0.07–1.31)	0.02	0.72	0.27
PPNA	Atrazine	N ≥ LOQ (%)	2 (7)	0	0.50	1 (7)	2 (13)	1.00	1.00	0.11
		Median (min–max)	0.14 (0.06–0.21)		na	0.16	0.08 (0.05–0.10)	0.22	1.00	na
	Bitertanol	N ≥ LOQ (%)	1 (4)	3 (10)	0.50	0	1 (7)	1.00	1.00	1.00
		Median (min–max)	0.20	0.14 (0.11–1.00)	0.66		0.10	na	na	0.18
	Carbendazim	N ≥ LOQ (%)	1 (4)	10 (33)	0.008	0	11 (73)	0.002	1.00	0.03
		Median (min–max)	0.37	0.29 (0.09–1.35)	0.53		0.35 (0.09–6.39)	na	na	0.46
	Methabenzthiazuron	N ≥ LOQ (%)	0	6 (20)	0.03	0	2 (13)	0.50	na	0.70
		Median (min–max)		0.15 (0.07–0.94)	na		0.07 (0.05–0.08)	na	na	0.10
	Metoxuron	N ≥ LOQ (%)	0	9 (30)	0.008	0	3 (20)	0.25	na	0.72
		Median (min–max)		0.15 (0.09–0.35)	na		0.10 (0.08–0.12)	na	na	0.11
Total exposure to pesticides	Σfmol_pest_/mg hair	Median (min–max)	12.5 (1.83–68.7)	68.7 (17.1–280)	<0.001	16.8 (3.35–173)	82.6 (22.9–571)	<0.001	0.19	0.31

^a^ = McNemar exact test. ^b^ = Wilcoxon test. ^c^ = Fisher exact test. ^d^ = Mann-Whitney test. na = not applicable.
